# Conformational analysis of flephedrone using quantum mechanical models

**DOI:** 10.1007/s00894-012-1673-z

**Published:** 2012-12-14

**Authors:** Wojciech Kolodziejczyk, Jerzy Jodkowski, Tiffani M. Holmes, Glake A. Hill

**Affiliations:** 1 Department of Physical Chemistry, Wroclaw Medical University, pl. Nankiera 1, 50-140 Wroclaw, Poland; 2 Department of Chemistry, Fort Valley State University, Fort Valley, GA 31030 USA; 3 Interdisciplinary Center for Nanotoxicity, Jackson State University, Jackson, MS 39217 USA; 4 Academic Classroom and Laboratory Bldg., Fort Valley State University, Room 206, 1005 State University Dr., Fort Valley, GA 31030 USA

**Keywords:** Conformations, Flephedrone, Molecular electrostatic potential

## Abstract

Flephedrone is an analogue of cathinone - chemically similar to ephedrine, cathine and other amphetamines. Conformations of all isomers of flephedrone have been studied at the quantum-chemical level. Calculations have been performed using DFT and MP2 methods with two basis sets - 6-31G and 6-31G(d,p). Results show that there are low energy conformers for the ortho, meta, and para isomers that are connected by way of low-barrier transition states. Boltzmann distribution of population predicts the highest population for the 1-meta conformer with a 10 % increase in solution. The molecular electrostatic potential surface data for each molecule has been calculated revealing likely reaction sites.

**Electronic supplementary material:**

The online version of this article (doi:10.1007/s00894-012-1673-z) contains supplementary material, which is available to authorized users.

## Introduction

In recent years, there has been a rapid development of “legal highs” - various psychotropic substances being sold and distributed around the world via the internet. Initially, these substances were freely available to buy legally and without restriction as fertilizers and incense and could be purchased using the internet or from “legal high” shops throughout the UK and Ireland [1, 2]. In 2010, the UK government responded to concerns about the safety of the drugs by banning their use, supply, or possession in the UK. The governments of other countries immediately followed with similar action, but there are still countries where one can buy them legally. Although there is no extensive scientific data on the structure of some of these psychoactive compounds, it is easy to find some basic information. The method of synthesis is easily accessed through web based sites and forums that are specifically designed to provide users means of acquiring “legal high” drugs. Because this information is so readily available to the general public, there is a real danger of overdose or poisoning from consumption. Since only a few of these compounds have been scientifically investigated [1–4] there exists a need to identify structural properties, determine the mechanism of action, and elucidate toxicological effects.

**Fig. 1:**
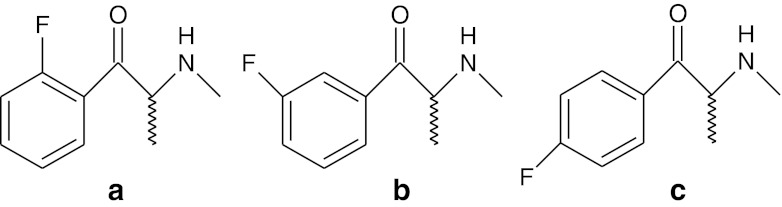
Structure of **a** 2-fluoromethcathinone (2-FMC, ortho) **b** 3-fluoromethcathinone (3-FMC, meta) **c** 4-fluoromethcathinone (4-FMC, para)

One such compound that produces psychoactive effects is flephedrone (4-fluoromethcathinone) (Fig. 1) [1, 2] which is an analogue of cathinone [4, 5]. Cathinone is an alkaloid found in the shrub Catha edulis (khat). It is chemically similar to ephedrine and other amphetamines. Flephedrone is easily synthesized from ephedrine [6], and it is believed that cathinone induces the release of dopamine from striatal preparations that are pre-labeled either with dopamine or its precursors [5].

The primary aim of this study is to identify all possible conformers of flephedrone so that these structures may be compared to the cathinone structures. There are two additional isomers of fluoromethcathinone which are 2-fluoromethcathinone and 3-fluoromethcathinone (Fig. 1). These isomers with 4-fluoromethcathinone are considered and calculated using ab initio and density functional treatments. Additionally, the molecular electrostatic potential isosurfaces (MEPS) are calculated.

## Computational methods

Potential energy surfaces were scanned, and all minima were optimized to find all possible conformers of cathinone, norephedrine and norpseudoephedrine. All structures have been obtained using a relaxed dihedral angle scan to generate a potential energy surface. Points were then optimized using density functional theory’s [7] B3LYP [8, 9] hybrid functional with 6-31G and 6-31G(d,p) basis sets. Second-order Moller Plesset (MP2) [10] calculations were then performed on the generated (DFT/B3LYP) surfaces. The thermodynamic parameters of all compounds were calculated using statistical mechanics expressions.

Solvation free energies (ΔGsolv) were determined at the DFT/B3LYP level with COSMO [11]. The ΔGsolv values were calculated in aqueous continuum (ε = 78.39), and the molecular cavities were built using the united atom model for Hartree–Fock (UAHF). In principle, the free energy surfaces are determined by a rigorous combination of free energy perturbation/umbrella sampling approaches [12, 13]. These surfaces reflect nonequilibrium solvation. However, such calculations are very challenging when performed within the ab initio framework. Thus, a more practical, simplified approach has been made. The relative free energies (ΔG) are determined as [14]:

1 $$ \varDelta \mathrm{G}=\varDelta {{\mathrm{H}}_{\mathrm{gas}}}\left( {298\mathrm{K}} \right)-\mathrm{T}\varDelta \mathrm{S}-\mathrm{RTln}\left( \omega \right)+\varDelta \varDelta {{\mathrm{G}}_{\mathrm{solv}}}\approx \varDelta {{\mathrm{G}}_{\mathrm{solv}}}\left( {298\mathrm{K}} \right)+\varDelta \varDelta {{\mathrm{G}}_{\mathrm{solv}}}. $$

In Eq. (1), ΔH_gas_(298 K) is enthalpy at 298 K, ΔS is the gas-phase entropy, ΔG_gas_ (298 K) is the Gibb’s free energy at 298 K, and ΔG_solv_ is the relative free energy of solvation. The contribution of the RT ln (ω) term is zero, as the electronic degeneracy term, ω, for the singlet state is unity. The ΔG values are used to calculate the relative population of the various conformers of the molecules under study in aqueous medium.

Since the molecules are much less rigid in solution than in the gas phase, we have used another definition of free energy, (Δg_flex_), to compare the intrinsic flexibility of different conformers, when embedded in aqueous medium. This is the practical implementation of the more general expression used by Warshel and coworkers [14, 15]. The Δg_flex_ could be expressed as:

2 $$ \varDelta {{\mathrm{g}}_{\mathrm{flex}}}=\varDelta {{\mathrm{E}}_{\mathrm{solute}}}+\varDelta \mathrm{ZPE}+\varDelta \varDelta {{\mathrm{G}}_{\mathrm{solv}}}-\alpha \mathrm{T}\varDelta \mathrm{S}, $$

where ΔE_solute_ and ΔZPE in Eq. (2) are the relative gas-phase energy separations and the zero-point energies of the various conformers. The scale factor α is usually taken as zero [15], and expression (2) can be simplified as:

3 $$ {\psi_{\mathrm{gflex}}}=\varDelta {{\mathrm{H}}_0}^{\mathrm{gas}}+\varDelta \varDelta {{\mathrm{G}}_{\mathrm{solv}}}. $$

All terms in Eqs. (1) and (3) are available from the thermochemical analyses based on statistical mechanics expressions using the ideal gas, rigid rotator, and harmonic oscillator approximations [16]. All calculations were carried out using the GAUSSIAN 03 structure calculation software [17], and molecular graphics were generated using the GAUSS VIEW visualization program [18].

## Results and discussion

### 2-FMC

**Fig. 2:**
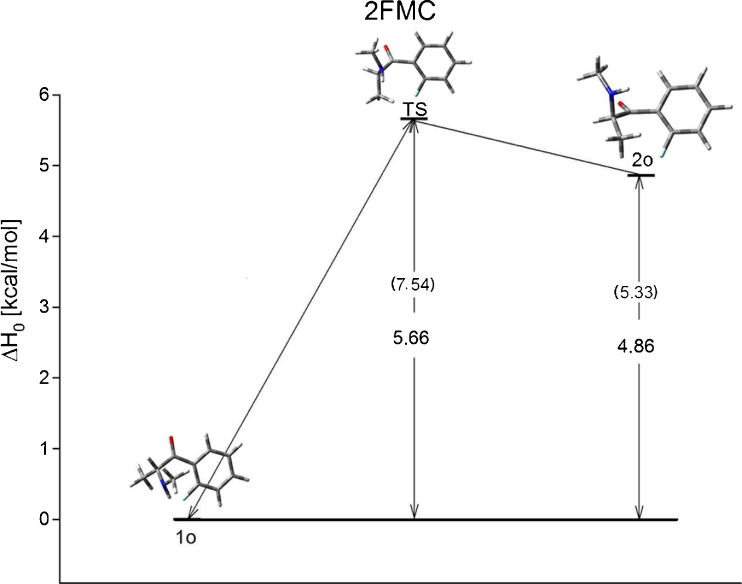
Energy profile for 2-FMC in gas phase. Energies are given in kcal mol^−1^. Energies in solution (g_flex_) are given in parenthesis

Results show that 2-FMC, 3-FMC and 4-FMC have two, four, and two low energy conformers, respectively. Thermodynamic and structural properties of 2-FMC have been collected in Table 1 and include relative enthalpy and free energy changes as well as angles of rotation. The two conformers have a 2.64 to 4.86 kcal mol^−1^ difference in ΔH_0_ depending on the basis set and method. Analysis of the structures shows that the structural properties of the conformers do not vary much with the changing of basis sets or method. Figure 2 is the energetic profile for the 2-FMC isomer with two conformers connected through a rotational transition state. The barrier for transition is equal to 5.66 kcal mol^−1^ for gas phase and 7.54 kcal mol^−1^ in aqueous solution.

**Table 1:** Relative energy and structural properties of low energy isomers of 2-FMC calculated at DFT and MP2 levels with 6-31G and 6-31G(d,p) basis sets. All energies are given in kcal mol^−1^

	6-31G		6-31G(d,p)						
2-FMC	ΔH_0_	ΔG	ΔH_0_	ΔG	τ_1_	τ_2_	τ_3_	μ	
DFT									
1o	0.00	0.00	0.00	0.00	39.5	22.3	179.5	3.62	6-31G
					40.7	22.6	179.1	3.25	6-31G(d,p)
2o	4.86	4.42	3.94	3.31	79.8	71.0	177.8	3.62	6-31G
					78.1	73.1	177.7	3.03	6-31G(d,p)
MP2									
1o	0.00	0.00	0.00	0.00	27.7	37.3	179.2	3.67	6-31G
					29.3	36.3	179.3	3.47	6-31G(d,p)
2o	3.16	3.32	2.64	2.72	79.8	71.0	177.8	3.99	6-31G
					70.6	89.4	178.4	3.22	6-31G(d,p)

### 3-FMC

**Fig. 3:**
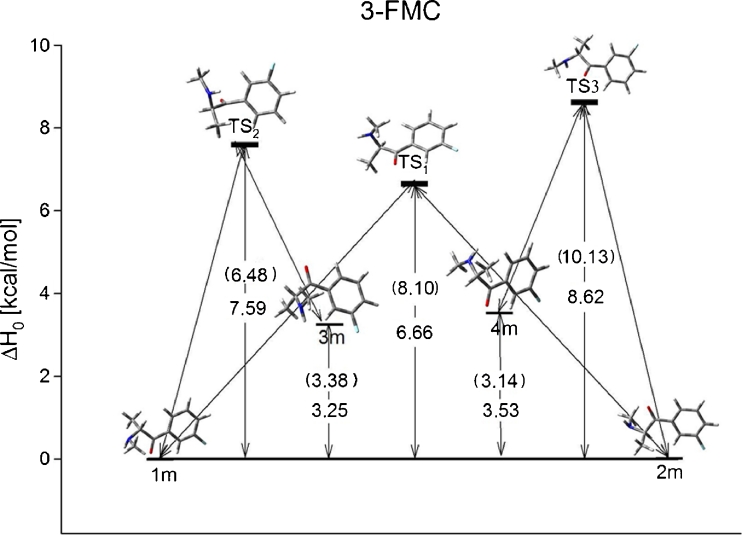
Energy profile for 3-FMC conformers in gas phase. Energies in solution (g_flex_) are given in parenthesis

Thermodynamic and structural parameters of 3-FMC isomers have been collected in Table 2 with four low energy conformers connected through five transition states (Fig. 3). Calculations using larger basis sets show that relative energy differences for each conformer increase. MP2 calculations demonstrate lower differences between conformers than the DFT results. Changing basis set or method does not have a significant effect on structural properties. Calculations in solution do not change relative differences between conformers, but it lowers the energy barrier for the transition of 1 m<−>2 m (Fig. 3). The Boltzmann distribution of population in Table 3 shows that the conformation with the highest population is the 1 m. In solution its population is increased by about 10 %. The higher dipole moment can contribute to the stabilization of the 1 m structure and influence the reactivity of the molecule in solution. Changing methods does not change the population percentages.

**Table 2:** Relative energy and structural properties of isomer of 3-FMC calculated using DFT and MP2 with 6-31G and 6-31G(d,p) basis sets. All energies are given in kcal mol^−1^

	6-31G		6-31G(d,p)						
3-FMC	ΔH_0_	ΔG	ΔH_0_	ΔG	τ_1_	τ_2_	τ_3_	μ	
DFT									
1 m	0.00	0.01	0.00	0.00	−157.1	−176.3	−179.3	3.38	6-31G
					−156.9	−175.9	−179.7	2.87	6-31G(d,p)
2 m	0.02	0.00	0.11	0.08	−157.2	3.6	179.9	1.36	6-31G
					−156.9	4.1	179.9	1.60	6-31G(d,p)
3 m	3.25	1.35	4.32	4.32	52.2	−1.1	178.9	1.58	6-31G
					49.9	16.9	179.3	1,83	6-31G(d,p)
4 m	3.53	2.89	4.28	4.26	52.6	−172.6	−179.3	4.29	6-31G
					50.4	−164.3	−179.8	3.31	6-31G(d,p)
MP2									
1 m	0.00	0.00	0.00	0.00	−161.7	−169.2	−179.6	3.20	6-31G
					−160.2	−172.2	−179.7	2.75	6-31G(d,p)
2 m	0.03	0.00	0.11	0.07	−161.7	12.8	179.5	1.31	6-31G
					−160.2	9.6	179.7	1.54	6-31G(d,p)
3 m	3.22	3.31	3.79	3.93	41.2	29.4	178.9	1.69	6-31G
					41.0	29.5	179.1	1.78	6-31G(d,p)
4 m	3.29	3.37	3.75	3.87	41.5	−152.1	−179.4	3.75	6-31G
					41.4	−152.1	−179.8	3.09	6-31G(d,p)

**Table 3:** Boltzman distribution of all isomers in gas phase and in solution calculated on DFT and MP2 level with 6-31G and 6-31G(d,p) basis set for 3-FMC isomer

	DFT		MP2	
	6-31G	6-31G(d,p)	6-31G	6-31G(d,p)
	Population [%]			
Gas phase				
1 m	50.67	54.59	51.05	54.53
2 m	48.99	45.33	48.53	45.28
3 m	0.21	0.04	0.22	0.09
4 m	0.13	0.04	0.20	0.10
Solvent				
1 m	64.79	66.32	66.52	52.65
2 m	34.67	33.30	31.95	46.70
3 m	0.21	0.12	0.49	0.28
4 m	0.32	0.26	1.04	0.37

### 4-FMC

**Fig. 4:**
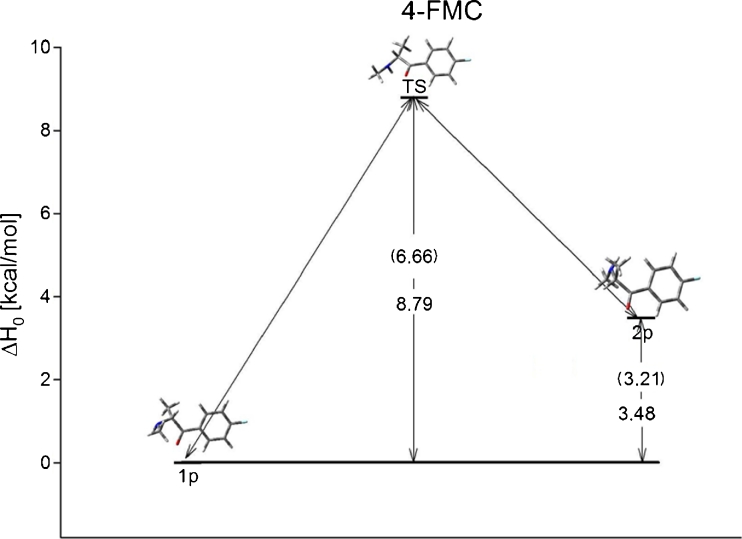
Energy profile for 4-FMC in gas phase. Energies in solution (g_flex_) are given in parenthesis

**Fig. 5:**
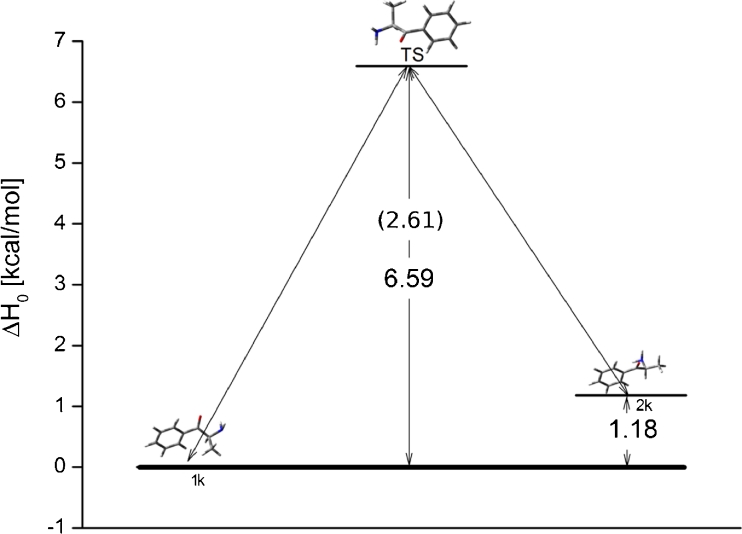
Energy profile for cathinone in gas phase. Energies are given in kcal mol^−1^. Energies in solution (g_flex_) given in parenthesis

The 4-FMC isomer has two conformers connected via one rotational transition state. Calculations using MP2 and DFT methods with 6-31G and 6-31G(d,p) basis sets show the same energetic trend for each conformer, although MP2 calculations predict lower energies. Table 4 contains all calculated structural and energetic properties of 4-FMC conformers with a graphical representation of each conformer shown in the Supporting information. Structural properties do not change much with higher basis set or different method of calculation. The rotational barrier for gas phase is equal to 8.78 kcal mol^−1^ for the lowest energy conformer (Fig. 4). In solution, this difference decreases to 6.66 kcal mol^−1^. The energy trend for the gas phase shown in Fig. 4 is the same in solution. The energy difference for conformers is between 3.5 kcal mol^−1^ and 4 kcal mol^−1^ depending on the basis set and method for the gas phase. Solution decreases the energy by about 0.20 kcal mol^−1^. Of the three isomers, cathinone has an energetic profile most similar to 4-FMC (Fig. 5). Free energy differences between conformers of cathinone and 4-FMC are from 0.98 to 1.71 kcal mol^−1^ while other isomer conformations have energy differences 1.5 to 3.5 kcal mol^−1^ higher (Table 5).

**Table 4:** Relative energy and structural properties of 4-FMC calculated using DFT and MP2 with 6-31G and 6-31G(d,p) basis sets. All energies are given in kcal mol^−1^

	6-31G		6-31G(d,p)						
4-FMC	ΔH_0_	ΔG	ΔH_0_	ΔG	τ_1_	τ_2_	τ_3_	μ	
DFT									
1p	0.00	0.00	0.00	0.00	−157.0	3.3	180.0	2.48	6-31G
					−160.0	3.8	180.0	2.12	6-31G(d,p)
2p	3.48	3.76	3.30	4.15	51.37	8.6	179.1	1.52	6-31G
					51.5	14.4	179.3	1.69	6-31G(d,p)
MP2									
1p	0.00	0.00	0.00	0.00	157.0	3.3	180.0	2.18	6-31G
					160.2	9.6	179.7	1.79	6-31G(d,p)
2p	3.30	3.41	3.81	3.95	51.4	8.6	179.1	1.20	6-31G
					41.6	28.6	179.1	1.42	6-31G(d,p)

**Table 5:** Relative energy and structural properties of low energy isomers of cathinone calculated at DFT and MP2 levels with 6-31G and 6-31G(d,p) basis sets. All energies are given in kcal mol^−1^

	6-31G		6-31G(d,p)						
Cathinone	ΔH_0_	ΔG	ΔH_0_	ΔG	τ_1_	τ_2_	τ_3_	μ	
DFT									
1 k	0.00	0.00	0.00	0.00	−179.8	175.8	158.2	3.09	6-31G
					−179.9	175.1	157.3	3.10	6-31G(d,p)
2 k	1.18	0.98	1.31	1.12	−179.6	7.1	78.4	3.11	6-31G
					−179.7	6.9	81.0	2.99	6-31G(d,p)
4-FMC									
MP2									
1 k	0.00	0.00	0.00	0.00	179.2	166.3	165.4	3.67	6-31G
					179.5	156.3	166.4	3.36	6-31G(d,p)
2 k	1.68	1.71	1.34	1.40	179.3	19.4	66.2	3.66	6-31G
					179.1	25.1	66.9	3.22	6-31G(d,p)

### Molecular electrostatic potential surface analysis

**Fig. 6:**
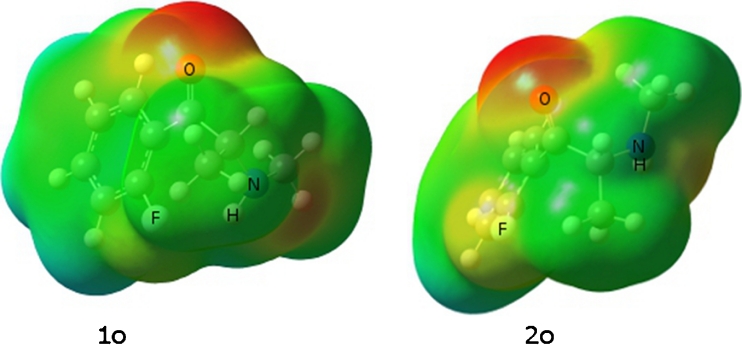
Molecular electrostatic potential surface of 2-FMC conformers. Red regions of the map are the most electron-rich regions of the molecule and blue regions are electron-poor. Order of increasing electron density is blue < green < yellow < orange < red

Molecular electrostatic potential maps (MEP) use local electronic charge density to systematically determine a molecule’s ability to interact. Widely used in drug design, MEP maps provide a method for identification of ligand-protein attractive forces. Generation of MEP maps for this study is ideal for predicting the nature of interactions and provides justification for molecular phenomena. The molecular electrostatic potential isosurface (MEP) for 2-FMC shows that the most active site of the molecule for reaction is on the oxygen atom (Fig. 6). The second negative region is localized around the nitrogen atom of the amine group. The negative potential zone around the oxygen atom is much stronger than for fluorine. Oxygen’s high potential is expected due to lone pair electrons, but nevertheless provides a primary site for strong hydrogen bonding. The active conformers have positioning of the nitrogen as a secondary amine group. This restricts the ability of nitrogen to give a strong negative potential.

**Fig. 7:**
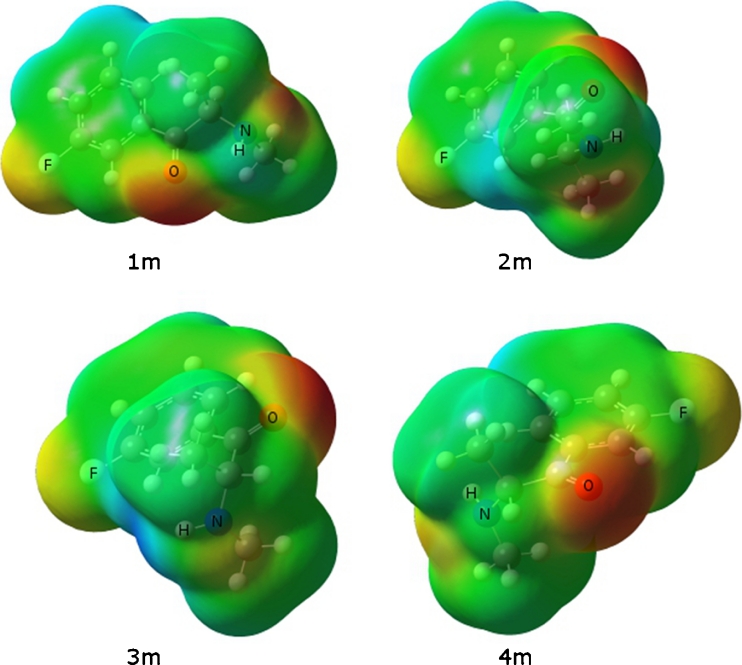
Molecular electrostatic potential surface of 3-FMC conformers. Red regions of the map are the most electron-rich regions of the molecule and blue regions are electron-poor. Order of increasing electron density is blue < green < yellow < orange < red

**Fig. 8:**
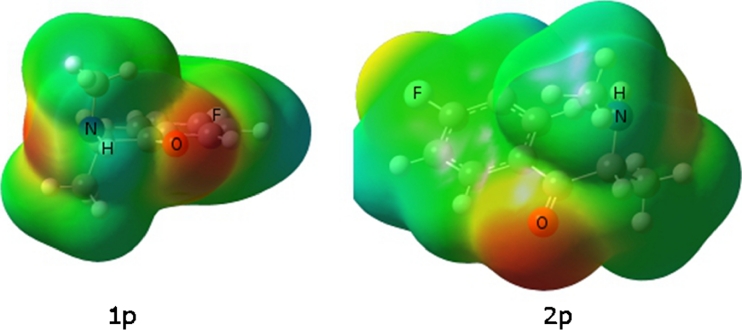
Molecular electrostatic potential surface of conformers of the 4-FMC isomer. Red regions of the map are the most electron-rich regions of the molecule and blue regions are electron-poor. Order of increasing electron density is blue < green < yellow < orange < red

Figure 7 is the MEP isosurface of all low-energy conformers of 3-FMC. The negative potential region is maximized around the oxygen atom. Because of the shape of the molecule there appears to be one more active region of negative potential on the fluorine atom, but the negative potential is much less negative than for the oxygen atom. For structure 1 m, the nitrogen atom also has negative potential. Figure 8 gives the MEP for the 4-FMC conformers. As with the conformers of 2-FMC and 3-FMC, areas of potential exist near the oxygen, nitrogen, and fluorine with the higher electron density around oxygen. As compared to FMC isomers, cathinone will possibly form a stronger N----H bond, but FMC’s higher number of potential binding sites will make FMC a competitive receptor ligand.

## Conclusions

All isomers have at least two low energy conformers connected through low-barrier transition states. In this work we carried out conformational analysis of isomers of fluoromethcathinone at the DFT-B3LYP/6-31G, B3LYP/6-31G(d,p), MP2/6-31G and MP2/6-31G(d,p) level. The dependence of the low-energy conformers of fluoromethcathinone on aqueous solvation was studied using the DFT|MP2/COSMO model and the MEP surfaces were generated for these low-energy surfaces to interpret the nature of practical reaction regions. Our calculations indicate that 2-FMC, 3-FMC, and 4-FMC have several low-energy conformers in the gas phase as well as in aqueous medium. These low-energy conformers are connected through low-barrier transition states, indicating that they can be converted from one form to the other. The energy trend is conserved for MP2/DFT calculations. In one case, (3-FMC) in aqueous medium, one transition barrier has been lowered in comparison with the gas phase. The MEP surfaces revealed three negative potential regions that can take place in reaction. Because of the geometry of these compounds there are few structures where the negative potential around nitrogen is weak. In closing, this study reveals the importance of low-energy conformers of flephedrone in exhibiting its specific biological properties. Obvious sites of reactivity exist which can participate in hydrogen bonding or charge transfer interactions. This implies that flephedrone, as with other psychostimulants, can act as an exogenous receptor agonist by receptor binding and indirect or direct activation. Understanding the structure offers a starting point for determining the mechanism of action and provides for the development of antagonist drugs as treatment of overdose, poisoning, or addiction.

## Electronic supplementary material

Below is the link to the electronic supplementary material.

ESM 1 (DOC 136 kb)

## References

[1] Archer RP (2009). Fluoromethcathinone: a new substance of abuse. Forensic Sci Int.

[2] Brandt SD, Sumnall HR, Measham F, Cole J (2010). Analysis of NRG ‘legal highs’ in the UK: identification and formation of novel cathinones. Drug Test Anal.

[3] Meyer MR, Wilhelm J, Peters FT, Maurer HH (2010). Beta-keto amphetamines: studies on the metabolism of the designer drug mephedrone and toxicological detection of mephedrone, butylone, and methylone in urine using gas chromatography–mass spectrometry. Anal Bioanal Chem.

[4] Zelger JL, Schorno X, Carlini EA (1980). Behavioural effects of cathinone, an amine obtained from Catha edulis Forsk: comparisons with amphetamine, norpseudoephedrine, apomorphine and nomifensine. Bull Narc.

[5] King L (2003). The misuse of drugs act: a guide for forensic scientist 2003.

[6] Belhadj-Tahar H, Sadeg N (2005). Methcathinone: a new post-industrial drug. Forensic Sci Int.

[7] Parr RG, Yang W (1989). Density functional theory of atoms and molecules.

[8] Becke AD (1993). Density–functional thermochemistry. III. The role of exact exchange. J Chem Phys.

[9] Lee C, Wang WP, Parr RG (1988). Development of the Colle-Salvetti correlation energy formula into a functional of the electron density. Phys Rev B.

[10] Moller C, Plesset MS (1934). Note on an approximation treatment for many electron systems. Phys Rev.

[11] Barone V, Cossi M (1998). Quantum calculation of molecular energies and energy gradients in solution by a conductor solvent model. J Phys Chem A.

[12] Hwang JK, King G, Creighton S, Warshel A (1988). Simulation of free energy relationships and dynamics of SN2 reactions in aqueous solution. J Am Chem Soc.

[13] Warshel A (1991). Computer modeling of chemical reactions in enzymes and solutions.

[14] Florián J, Warshel A (1998). Phosphate ester hydrolysis in aqueous solution: associative versus dissociative mechanisms. J Phys Chem B.

[15] Florián J, Štrajbl M, Warshel A (1998). Conformational flexibility of phosphate, phosphonate, and phosphorothioate methyl esters in aqueous solution. J Am Chem Soc.

[16] Davidson N (1962). Statistical mechanics.

[17] Frisch MJ, Trucks GW, Schlegel HB, Scuseria, GE, Robb MA, Cheeseman, JR, Montgomery JA Jr, Vreven T, Kudin KN, Burant JC, Millam JM, Iyengar SS, Tomasi J, Barone V, Mennucci B, Cossi M, Scalmani G, Rega N, Petersson GA, Nakatsuji H, Hada M, Ehara M, Toyota K, Fukuda R, Hasegawa J, Ishida M, Nakajima T, Honda Y, Kitao O, Nakai H, Klene M, Li X, Knox JE, Hratchian HP, Cross JB, Bakken V, Adamo C, Jaramillo J, Gomperts R, Stratmann RE, Yazyev O, Austin AJ, Cammi R, Pomelli C, Ochterski JW, Ayala PY, Morokuma K, Voth, GA, Salvador P, Dannenberg JJ, Zakrzewski VG, Dapprich S, Daniels AD, Strain MC, Farkas O, Malick DK, Rabuck AD, Raghavachari K, Foresman JB, Ortiz JV, Cui Q, Baboul AG, Clifford S, Cioslowski J, Stefanov BB, Liu G, Liashenko A, Piskorz P, Komaromi I, Martin RL, Fox DJ, Keith T, Al-Laham MA, Peng CY, Nanayakkara A, Challacombe M, Gill PM W, Johnson B, Chen W, Wong MW, Gonzalez C, Pople JA (2004) Gaussian 03. Gaussian Inc, Pittsburgh, PA

[18] Commercial Molecular Graphics Software (2005) GaussView 2005. Gaussian Inc, Pittsburgh, PA

