# Supplementary material for: Conformational analysis of flephedrone using quantum mechanical models
Source: J Mol Model. 2012 Dec 14;19(3):1451–8. doi: 10.1007/s00894-012-1673-z (PMC3578731; doi:10.1007/s00894-012-1673-z)
Supplement: Supplementary file 1 — (DOC 136 kb) [file 894_2012_1673_MOESM1_ESM.doc]

Structure of cathinone


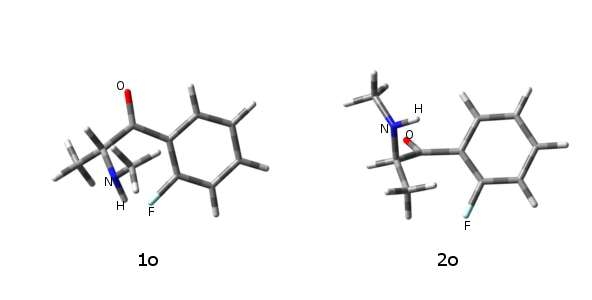


Conformations of 2-FMC


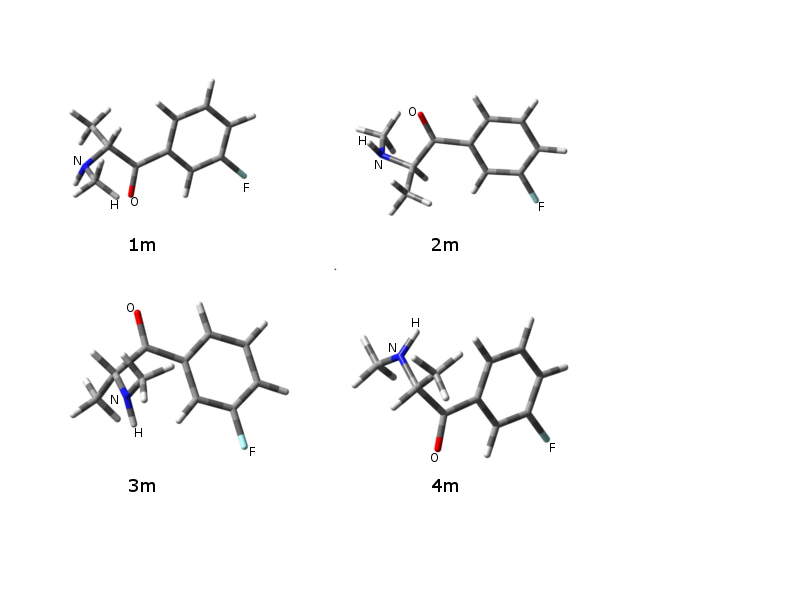


All structures of 3-FMC conformers


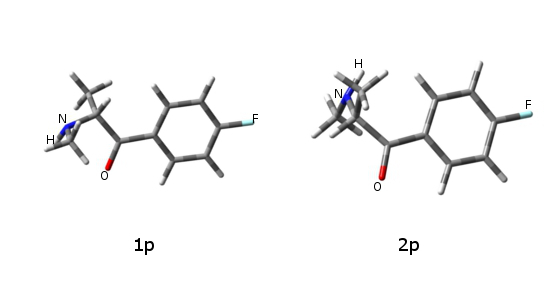


Structures of 4-FMC conformers.
